# OPTIMIZING A PRAGMATIC TRIAL OF EMERGENCY DEPARTMENT INITIATED CARE FOR PERSONS LIVING WITH DEMENTIA

**DOI:** 10.1093/geroni/igae098.1131

**Published:** 2024-12-31

**Authors:** Abraham Brody, Manish Shah, Keith Goldfeld, Corita Grudzen, Joshua Chodosh

**Affiliations:** New York University Rory Meyers College of Nursing, New York, New York, United States; University of Wisconsin School of Medicine & Public Health, Madison, Wisconsin, United States; New York University Grossman School of Medicine, New York, New York, United States; Memorial Sloan Kettering Cancer Center, New York, New York, United States; New York University Grossman School of Medicine, New York, New York, United States

## Abstract

Over 50% of persons living with dementia (PLWD) seek emergency department (ED) care annually. However, the ED is not optimized to care for PLWD; those discharged back to the community experience adverse events. Through a recently-funded NIA Cooperative Award, we will implement an 80-site embedded pragmatic factorial designed trial of three independent, yet potentially synergistic, interventions to improve care and reduce adverse events during care transitions among PLWD and their care partners who visit the ED: Emergency care redesign, nurse-led telephonic care, and community paramedic-led transitions. While each intervention has been previously implemented, they have not been implemented concurrently. Thus, to prepare for the full-scale trial where EDs will implement zero to all three interventions, we performed an optimization study with 40 PLWD who received care and were discharged from two EDs. The optimization program examined how to efficiently implement interventions in tandem while maintaining core functions. We developed and implemented: clinical decision support pathways to identify PLWD eligible for the clinical interventions and nudge clinicians towards effective intervention implementation, modifications for when multiple interventions were utilized, and intra-intervention communication pathways. We evaluated the optimization through implementation metrics and semi-structured interviews with engaged clinicians, PLWD and their care partners. Particular challenges requiring adaptations included time required to implement clinical decision support, creating effective communication pathways between intervention programs, developing staffing flexibility in the nurse telephonic program to allow for coverage at key times while balancing nursing staff needs, and engaging ED staff in performing team huddles at the bedside.

